# Exploring Capability, Opportunity and Motivation in Weight‐Related Communication: Service User and Staff Experiences in a UK‐Based Secure Mental Health Unit

**DOI:** 10.1111/inm.70400

**Published:** 2026-11-13

**Authors:** Novia K. N. Mak, Bridget Dibb, Nicholas Stokes, Elizabeth Alexandra Barley, Magdalena Zasada

**Affiliations:** ^1^ Faculty of Health and Medical Sciences, School of Health Science University of Surrey Guildford UK; ^2^ School of Psychology Elizabeth Fry Building (AD) University of Surrey Guildford, Surrey UK; ^3^ Research Department St Bernard's Hospital Southall UK

**Keywords:** communication, mental disorders, nursing staff, obesity, qualitative research

## Abstract

Excess weight and related health conditions are highly prevalent in secure mental health settings, yet engaging service users in weight‐related discussions remains challenging. Nursing staff are well placed to lead these conversations, but must navigate competing priorities, role boundaries and concerns about negative responses, while service users may experience cognitive and motivational difficulties. This study examined barriers and enablers to weight‐related discussions from the perspectives of service users and nursing staff within UK‐based low‐ and medium‐secure units. A qualitative design was used. Semi‐structured interviews were conducted with 20 service users and 10 nursing staff. Data were analysed using framework analysis informed by behavioural theory to identify factors influencing engagement in weight‐related communication. Findings showed that engagement was shaped by interacting individual, relational and organisational factors. Nursing staff identified communication skills, experience and access to resources as key enablers but reported barriers including limited training, competing demands and concerns about damaging therapeutic relationships. Service users described barriers including cognitive challenges, environmental restrictions and beliefs about medication‐related weight gain, alongside motivational factors such as emotional eating. Across both groups, relational dynamics, including trust, power and communication style, were central in determining whether discussions were initiated and sustained. Within this unit context, weight‐related discussions in secure mental health settings are not solely clinical tasks but relational practices. Improving engagement requires strengthening staff communication skills, creating supportive environments and embedding collaborative, personalised approaches within routine care. While findings reflect a single UK setting, the relational and structural mechanisms identified offer transferable insights for co‐designing physical health communication strategies across similar secure mental health services.

## 1 Introduction

Excess weight and obesity are disproportionately prevalent among individuals living with severe mental illness (SMI), and contribute to significantly higher rates of cardiovascular disease, diabetes and premature mortality (NHS England 2024). These health disparities are pronounced in secure mental health settings (Johnson et al. 2018). Eighty per cent of service users in the UK secure settings are overweight or obese (Public Health England 2021), with similarly high prevalences reported internationally across secure psychiatric services in Australia (96.6%), Europe (51.6%–94%) and America (72.4%) (Jeandarme et al. 2024; Ma et al. 2021; Moss et al. 2024).

Although the organisation of secure services varies internationally, many share common features, including restricted environments, reduced autonomy and prolonged inpatient stays, all of which contribute to obesogenic conditions and shape opportunities for physical health engagement (Mills et al. 2024; Zagham et al. 2024). Within UK low and medium‐secure units, service users experience multiple barriers to maintaining healthy lifestyles, including environmental restrictions (Rogers et al. 2021), reduced autonomy (Every‐Palmer et al. 2018), psychotropic medication side effects (Sass et al. 2024) and limited access to nutritious food and physical activity opportunities (McCullough 2023).

Despite growing policy attention to physical health in secure mental health care, weight management interventions are inconsistently implemented and rarely sustained in practice (Huthwaite et al. 2017). Weight is a sensitive issue, closely linked to identity, self‐esteem and recovery (Dandgey and Patten 2023). Nurses are well‐positioned to initiate and sustain these conversations due to their continuous therapeutic contact and responsibility for both physical and mental health care (Happell et al. 2023). However, both nurses and service users report discomfort and uncertainty around weight‐related discussions, which may be perceived as intrusive, judgemental, or professionally risky (Attala et al. 2022; Moyles et al. 2023). Understanding how these conversations are initiated and experienced from both perspectives is therefore critical to inform acceptable, co‐designed practice.

### 1.1 Background

Existing research on weight management in people with SMI has predominantly focused on the effectiveness of structured interventions, such as diet or exercise programmes (Day and Johnson 2017; Stevens et al. 2023). Far less attention has been given to the interpersonal and contextual processes that shape how weight‐related discussions are initiated, framed and sustained within therapeutic relationships, particularly in secure mental health settings. Moreover, much of the wider literature excludes people with SMI or does not account for the relational, environmental and power dynamics characteristic of secure care (Sagi‐Dain et al. 2022). To our knowledge, only one study has examined the perspectives of both service users and nursing staff on weight management in secure mental health settings (Prebble et al. 2011).

This study, therefore, explores how nursing staff and service users experience and navigate weight‐related discussions in secure mental health units. The analysis is informed by the COM‐B model, which conceptualises behaviour as arising from the interaction between capability, opportunity and motivation (Michie et al. 2011). This framework is well‐suited to examining weight‐related communication in secure settings, where engagement is shaped not only by knowledge but also by relational dynamics, environmental constraints and power asymmetries. As effective communication is a prerequisite for shared understanding and collaborative behaviour change (Albury et al. 2020; Michie et al. 2014), examining how these discussions are initiated and experienced addresses a critical gap in the literature.

High secure settings were excluded from this study. Although all secure settings share restrictive and potentially obesogenic features, high secure units involve greater levels of procedural and relational restriction and more limited access to facilities, which may differentially shape autonomy, physical activity and staff‐service user interaction (Duffy et al. 2024). These contextual differences support the need for separate investigation.

### 1.2 Research Question

- What are the views of service users and nursing staff on the perceived facilitators and barriers when initiating and engaging in weight‐related discussions within a UK secure mental health units?

## 2 Methods

### 2.1 Design

A qualitative design, informed by the Theoretical Domains Framework (TDF) and the COM‐B model (Atkins et al. 2017; Michie et al. 2011), was adopted.

#### 2.1.1 Setting

The study was conducted within an NHS mental health inpatient setting in England. The study site encompassed 18 wards: six low‐secure inpatient wards (five male and one female) and 12 medium‐secure inpatient wards (seven male and five female). Participants were recruited from a subset of these wards, selected to capture a range of security levels, gender distribution and clinical profiles. The research site population comprised 246 service users (inpatients), and approximately 210 qualified nursing staff and 188 healthcare facilitators in the inpatient forensic service at the time of this study. The service users in these units are typically diagnosed with SMI and experience disproportionately high rates of obesity, presenting unique challenges to weight management. Recent data collected by the hospital's research department in June 2024 indicated that the overall obesity rate within the Hospital is 53.3%, with 13.1% of service users classified as severely obese and 0.9% as underweight. Only 11.7% fall within the healthy BMI range, while a total of 74.3% of service users are classified as overweight or obese (N. Stokes, personal communication, 23 April 2024). The prevalence of obesity in this setting underscores the clinical significance of weight management interventions and highlights the need to understand the barriers and enablers that influence their implementation. The selected NHS Trust was a collaborative partner in the study and facilitated access to the research site.

#### 2.1.2 Sampling and Recruitment

A purposive sampling approach was used to recruit nursing staff and service users with varied experiences and characteristics relevant to the research question.

##### 2.1.2.1 Nursing Staff

Recruitment was facilitated through collaboration with ward managers and clinical leads. Study information sheets and invitations were circulated via staff email and displayed on ward noticeboards. The researcher also attended team meetings to introduce the study, answer questions and allow staff to express interest voluntarily.

##### 2.1.2.2 Service Users

Recruitment of service users was supported by the nursing team, who provided an accessible information sheet and obtained verbal consent for the researcher to make contact. Researchers then met interested service users individually to explain the study in detail and obtain written informed consent. Participation was entirely voluntary. Service user participants received £11.95 per session, funded by the research site, in line with National Institute for Health and Care Research guidelines on remuneration for patient and public involvement (NIHR 2024). Confidentiality was emphasised throughout.

Inclusion criteria for service users:

- A diagnosis of SMIs
- Residing in a low or medium‐secure unit within the selected NHS Trust
- Judged by the clinical team to be able to reflect meaningfully on weight‐related health experiences
- Capacity to provide informed consent
- Sufficient English proficiency to participate in an interview

Inclusion criteria for nursing staff:

- Nursing staff registered with the Nursing and Midwifery Council (NMC), or healthcare assistants working in the selected NHS Trust low and medium‐secure units
- Directly involved in service user care
- Willing to reflect on their experiences discussing physical health and weight management

#### 2.1.3 Participants

Thirty people were recruited, comprising 20 service users (12 male, 8 female) and 10 nursing staff. Service users were predominantly from Black and minority ethnic backgrounds and represented both low and medium‐secure units. Nursing staff reflected a range of professional experience and ethnic backgrounds. Detailed demographic characteristics are presented in Tables 1 and 2.

**TABLE 1:** Demographic characteristics of service user participants (*n* = 20).

Characteristics	Categories	*n*
Gender	Female	8
	Male	12
Age group	24–35 years	10
	35–54 years	9
	> 54 years	1
Ethnic background	White	3
	Asian/Asian British	2
	Black/Black British/Caribbean/African background	12
	Mixed/Multiple ethnic groups	2
	Other	1
Primary diagnosis	Schizophrenia	9
	Bipolar disorder	5
	Schizoaffective disorder	2
	Severe depression	4
Level of security	Low secure	10
	Medium secure	10

Abbreviation: *n*, number of participants.

**TABLE 2:** Demographic characteristics of nursing staff participants (*n* = 10).

Characteristics	Categories	*n*
Gender	Female	5
	Male	5
Age group	< 24 years	1
	25–34 years	3
	35–54 years	4
	> 55 years	2
Ethnic background	White	1
	Asian/Asian British	4
	Black/Black British/Caribbean/African background	5
RMN qualification	RMN‐qualified	7
	Not RMN‐qualified	3
Year of RMN qualification (among RMNs)	1–3 years	1
	4–8 years	4
	> 8 years	2
Experience in forensic care	1–3 years	4
	4–6 years	2
	7–10 years	2
	> 10 years	2

Abbreviations: *n*, number of participants; RMN, Registered Mental Health Nurse.

### 2.2 Data Collection

Semi‐structured interviews were conducted between 1 November 2021 and 30 October 2022. Interview schedules were informed by the Theoretical Domains Framework (Atkins et al. 2017) and COM‐B (Michie et al. 2011) and were developed and refined iteratively through Patient and Public Involvement (PPI), with input from service users, nursing staff and the multidisciplinary team. This process was undertaken to ensure that the interview questions were relevant, accessible and sensitive to the context of secure mental health care (Mak et al. 2026).

PPI contributors advised on the interview topic guides, including the framing and sequencing of questions, use of appropriate language and areas requiring particular sensitivity, such as medication‐related weight gain and power dynamics within nurse‐service user relationships (Mak et al. 2026). The interview schedules were subsequently piloted and refined to improve clarity, conversational flow and depth of response. For example, ‘When was the last time you talked about weight?’ was revised to ‘Can you describe the last time you had a conversation about weight?’ Similarly, ‘Are you confident to talk about weight with patients here?’ was changed to ‘How confident do you feel talking about weight with service users here?’.

Feedback from nursing staff also informed the use of an initial question asking whether they discussed weight with service users before asking them to describe a specific conversation, thereby avoiding assumptions that such conversations had occurred. Service‐user feedback informed the use of conversational linking prompts, such as ‘Thinking about this…’, to reduce an overly interrogative style and encourage participants to elaborate on their experiences. Further details of the PPI and co‐design process have been reported elsewhere (Mak et al. 2026).

The interview schedule questions covered:

- Experiences and comfort levels when discussing weight and health behaviours
- Perceived facilitators and barriers
- Organisational and cultural influences on such discussions
- Recommendations for improvement

Interviews were conducted in private rooms within the units, lasted between 28 and 60 min, and were audio‐recorded with participants' consent. Recordings were transcribed verbatim and anonymised. Field notes were used to capture non‐verbal cues and contextual observations.

### 2.3 Ethical Considerations

A favourable ethical opinion was provided by the University Research Ethics Committee, the NHS Research Ethics Committee (Reference: SPON202003FHMS), with approval from the Health Research Authority (IRAS: 278190).

Given the sensitivity of discussing body weight and health behaviours, the researcher (N.M.) was trained in trauma‐informed interviewing and debriefed participants after each session.

### 2.4 Data Analysis

Framework analysis was used to analyse the data using the five stages described by Gale et al. (2013): familiarisation, identifying a thematic framework, indexing, charting and mapping/interpretation.

Initial coding was conducted inductively to capture participants' personal meanings and experiences. These codes were then organised deductively using the Theoretical Domain Framework (TDF) (Atkins et al. 2017) and the COM‐B model (Michie et al. 2014). The COM‐B model helped situate findings within the three core components of behaviour change: capability, opportunity and motivation, while the TDF enabled deeper analysis of the specific cognitive, social and environmental factors underlying these domains. Using these frameworks in combination strengthened both the rigour and practical relevance of the analysis, allowing findings to be directly applied to the design of future nurse‐led weight management interventions in secure mental health settings.

To enhance rigour and consistency of the coding process, three transcripts were independently coded by two researchers (N.M., with a nursing background, and C.G., with a psychology background), under the supervision of experienced qualitative researchers: E.B., the former primary supervisor of this research project, a professor with both a nursing background and expertise as a health psychologist, and B.D., the second supervisor, a health psychologist. Both have extensive experience in qualitative research. Their collective expertise ensured the rigour and consistency of the coding process, and discrepancies were resolved through supervision.

In the final analysis stage, the researchers (N.M., C.G., E.B. and B.D.) identified the most prominent theoretical domains. Identifying these prominent domains is a standard practice within the TDF, as it informs the development of interventions targeting the most significant factors influencing professional and service user behaviour (Atkins et al. 2017). Prominent domains were identified based on criteria used in previous studies, which defined a domain as prominent if (a) participants frequently agreed on its importance or (b) the domain was discussed in depth or over a prolonged period (Johnston et al. 2022).

### 2.5 Rigour

Rigour was addressed through reflexive practice and application of established criteria of trustworthiness: credibility, dependability, confirmability and transferability (Ahmed 2024). Triangulation across participant groups and supervisory oversight supported the credibility and transparency of the analysis.

## 3 Results

A range of behavioural, interpersonal and organisational factors influenced the initiation and quality of weight‐related discussions. Findings were organised under the COM‐B domains of capability, opportunity and motivation, encompassing nursing staff, service users and shared perspectives. Key themes are summarised in Table 3.

**TABLE 3:** Summary of key barriers and enablers to weight‐related discussions in secure mental health inpatient settings, mapped to COM‐B domains from nursing staff and service users' perspectives.

Participant group	Thematic domains and subthemes
3.1 Nursing staff	3.1.1 Capability: Skills and Knowledge 3.1.2 Opportunity: Ward Culture and Power Dynamics 1.3 Motivation: Role Perception and Outcome Expectancies
3.2 Service user	3.2.1 Capability: Knowledge and Cognitive Challenges 3.2.2 Opportunity: Social and Organisational Environment 3.2.3 Motivation: Beliefs and Emotional Factors
3.3 Shared themes	3.3.1 Environmental Constraints 3.3.2 Power, Autonomy and Relational Safety 3.3.3 Need for Personalisation and Collaboration

*Note:* Themes are organised according to the COM‐B model, where behaviour is understood as the interaction between capability, opportunity and motivation.

### 3.1 Nursing Staff Perspectives

#### 3.1.1 Capability: Skills and Knowledge

Nursing staff identified communication skills and clinical experience as key enablers of weight‐related discussions:

We bring the [weight‐related] topic to the service users…because it …encourages them to choose healthy food (NS2)

Some staff drew on broader clinical experience, suggesting that weight‐related discussions were not inherently more difficult than other sensitive conversation routinely held in secure care:

I feel like staff experience enough, they would have had to go and speak to patients about so many more sensitive things (NS1)

However, this confidence was not consistent across the workforce. Several nurses described limited training in behaviour change approaches, uncertainty about applying techniques such as motivational interviewing (Miller and Rollnick 2012), and concern about providing inaccurate advice. These factors acted as barriers to initiating or sustaining weight‐related discussions:

I personally feel that staff do not have that confidence in talking to our service user about weight management…I think it's a fear of the staff…there's so many conflicting information…I am not a dietician, so I may end up giving the wrong advice (NS10)

Competing clinical demands further constrained staff capability in practice, as health promotion discussions were often deprioritised amid acute care responsibilities. Overall, while interpersonal skills and experience enabled some staff to engage confidently, gaps in structured training and role clarity limited consistency in weight‐related discussions.

#### 3.1.2 Opportunity: Ward Culture and Power Dynamics

Opportunities to initiate and sustain discussions about weight management were strongly shaped by ward culture and organisational norms. A prevailing culture of caution often discouraged staff from raising weight‐related topics, particularly where concerns existed about distress or escalation:

Don't want to push a boundary because the patient gets agitated…and when they get agitated, they can become aggressive (NS10)

Staff described tension between their therapeutic and custodial roles, noting that advice about weight could be interpreted as authoritative or coercive if not carefully framed:

He refused to do what [I] asked, as if I was trying [to be] authority… you have to structure the conversation carefully (NS7)

Establishing trust and relational safety was therefore seen as a prerequisite for initiating discussions:

You have to build that trust because [to them], we're all here like spies (NS7)

Staff described strategies such as timing conversations when service users are calm, seeking permission to discuss weight, and explicitly emphasising therapeutic intent. These approaches enabled more open dialogue:

if it's okay to talk about this kind of thing… maybe no…if the patient is really unwell… if it's safe or ready or calm if it's comfortable (NS2)

Ward's cultural and relational context shaped whether discussions were initiated or avoided.

#### 3.1.3 Motivation: Role Perception and Outcome Expectancies

Nurses' motivation to engage in discussions about weight management was closely tied to their professional identity and perceived responsibility. Many participants recognised physical health as an essential component of their duty of care:

Physical health [is part of my] duty of care… we don't want anything bad happen to you, the plan is for you guys to get well, get discharged (NS1)

However, competing clinical priorities acted as a barrier. Some staff prioritised mental health stabilisation over physical health interventions, reflecting the complex demands within secure services:

They have mental health issues as well, so our priority here is mental health (NS6)

Emotional labour and anticipated negative reactions further reduced motivation. Staff described anxiety about damaging therapeutic relationships or being perceived as judgmental:

I don't open that conversation because … he or she feels that staff is attacking him with regard to their weight (NS6)

Concerns about complaints or allegations, particularly when discussing weight with female service users, reinforced avoidance:

It's a bit difficult when you talk to females regarding the weight…have been accused of … being abusive …so to prevent those things you're just…take a step backward… as soon as the allegation comes, we suspend you until we complete the investigation (…)…so you want to avoid all these inconveniences… so it just don't want to…start the conversation (NS8)

To manage these risks, staff adapted their approach by reframing discussions around general health, avoiding stigmatising language, and choosing moments when service users appeared settled:

I'm not trying to tell you to lose weight but we are trying to look after your health. (NS1)

They don't like to be discussed with others…something like, you're overweight…you don't use that word… it's quite a strong word… they feel…this is too much focused, that one's weight, so they wouldn't like … to discuss that one… somebody so observing or focusing on your weight (NS5)

I focus on the safety of the ward, our safety and their safety first… this morning we talk [about weight] when they're settled… if I don't hear any risks…it's a good time for me to talk to them about [weight] (NS5)

Together, these findings show that staff motivation was shaped by a balance of professional commitment, emotional risk and organisational context, influencing whether weight‐related discussions were initiated or deferred.

### 3.2 Service User Perspectives

#### 3.2.1 Capability: Knowledge and Cognitive Challenges

Service users described context‐specific capability barriers that affected their ability to engage meaningfully in weight‐related discussions within the secure setting. Rather than lacking general health knowledge, participants emphasised uncertainty about what was permitted, available or realistic in this environment:

I don't know how to stay healthy in this place (SU3)

What are the rules or regulations [in this setting] and how can I support myself…get healthy? (SU12)

Cognitive challenges, including memory deficits, reduced comprehension and impaired decision‐making, frequently attributed to medication effects, further hindered consistent engagement in ongoing weight‐related dialogue:

People with disabilities and conditions…they can't comprehend…you might not understand… how to decide what would have been [healthy option] (SU6)

My memory is not that good, it's not that good [because] of medication…if you told me yesterday what did you eat? I can't just put my fingers and then tell you what I ate (SU18)

Rather than rejecting discussions about weight, participants indicated that one‐off or information‐heavy conversations were difficult to retain, limiting continuity and follow‐up. Service users suggested that structured, repeated and clearly communicated discussions, adapted to individual attributes would be better support engagement over time.

#### 3.2.2 Opportunity: Social and Organisational Environment

Service users identified missed opportunities for weight‐related discussions, particularly during admission. A lack of early, proactive communication from staff about diet, physical activity and available resources left participants uncertain about weight gain risks and available support:

More information about… what kind of exercise you can do in here [secure setting]… what kind of special diet [that I can access] … is better to tell me when I first got here (SU15)

Inconsistent staff practices and poor role modelling further undermined the credibility of weight‐related discussions. When staff behaviour appeared misaligned with health messages, service users questioned the sincerity and legitimacy of conversations:

Some of the staff are overweight… so that's not setting… a good example (SU2)

If you are saying one thing to a patient … doing the opposite (SU3)

Conversely, visible staff engagement and shared participation created opportunities for informal, relational discussions about weight and health:

[Nursing staff] encouraged me …I'll be more inclined (SU18)

Staff also come to the gym with us …they interact with us (SU15)

These findings highlight how organisational practices shaped opportunities not only for activity, but for conversation and relational engagement around weight.

#### 3.2.3 Motivation: Beliefs and Emotional Factors

Service users' motivation to engage in weight‐related discussions was influenced by emotional experiences, beliefs about the inevitability of weight gain and perceived communication tone. Emotional eating, boredom and low confidence often disengaged engagement:

Not doing much in this space…makes you want to eat though you're full up (SU18)

When I am feeling low… I will just eat one box onto the next (SU12)

Beliefs about medication‐related weight gain reduced the perceived value of discussion:

Medication impacts energy levels… you lose confidence, especially if you have mental health [struggles] (SU18)

Importantly, communication style strongly shaped motivation. Service users consistently emphasised that the absence or quality of communication from nursing staff shaped whether weight‐related discussions felt possible, safe or worth engaging in. Several participants indicated that when communication was perceived as authoritarian, dismissive or infantilising, engagement in weight‐related discussions was actively undermined. One service user described how directive and disrespectful communication eroded trust and willingness to engage:

I am older than the nurses and they talk to me like I'm a child… they make orders like…with an aggressive type of tone (SU10)

In contrast, collaborative, non‐judgmental and personalised approaches were described as empowering rather than confrontational, helping to counter feelings of shame or futility:

You give yourself a …goal… it kept me motivated …working towards something (SU15)

Deal with the person more individually (SU12)

These accounts suggest that motivation to engage in weight‐related discussions depended less on individual attitudes and more on whether conversations were initiated early, framed collaboratively and conducted in a relationally safe and respectful manner.

## 4 Shared Environmental Factors and Relational Dynamics

While Sections 1 and 2 present distinct nursing staff and service user perspectives, this section synthesises shared themes, illustrating how environmental and relational conditions shaped the initiation, quality and sustainability of weight‐related discussions. Consistent with the COM‐B model, these shared dynamics primarily influenced opportunity (environmental and organisational conditions that enable or constrain discussion) and motivation (relational safety, power and autonomy), while also interacting with capability by influencing staff confidence and service users' willingness to engage. Rather than operating in isolation, environmental and relational factors functioned as the context within which weight‐related conversations were either facilitated or curtailed.

### 4.1 Environmental Factors

Both groups described how organisational conditions directly affected whether weight‐related discussions could occur. Access to on‐site gyms and dietitian input enabled staff to initiate more concrete and meaningful conversations grounded in available options. However, staff shortages, high acuity and rigid routines frequently curtailed time and relational space for discussion:

If we are short of staff… we are very busy…sometimes we can't approach [service users] (NS6)

Service users similarly experienced these structural pressures as limiting opportunities to raise questions or seek encouragement about weight‐related concerns:

It's difficult to ask a nurse to walk with me…because they're short of staff. (SU1)

These findings demonstrate that organisational conditions directly shaped whether weight‐related discussions could occur at all, highlighting how opportunity within the COM‐B framework operates not only through access to resources, but through the availability of time, continuity and relational presence.

### 4.2 Power and Autonomy

Power dynamics within secure units emerged as a shared influence on engagement in weight‐related discussions. Service users described experiencing some interactions as infantilising or authoritarian, which discouraged them from raising sensitive topics such as weight. These experiences reduced trust and willingness to engage in dialogue, particularly where conversations felt directive rather than collaborative. Staff similarly acknowledged the need to carefully structure conversations to avoid being perceived as coercive, highlighting how power and autonomy shaped both the initiation and reception of weight‐related discussions.

Conversely, when staff demonstrated empathy, respect and a non‐judgmental approach, it enabled engagement in weight‐related discussions. The relational style of the nurse, rather than the topic itself, was central to whether conversations felt acceptable:

I feel okay for her to address [weight management]… doesn't bother me. If it was another staff, it would have bothered me, because this staff is very gentle (SU4)

Mutual frustration also functioned as a barrier by shaping how power and autonomy were experienced within everyday interactions. Staff described feeling constrained by organisational pressures that limited their ability to follow through on agreed plans or sustain ongoing dialogue. Service users, in turn, interpreted this inconsistency as disempowering, reinforcing perceptions that decisions about their weight and health were system‐driven rather than collaboratively negotiated. These dynamics illustrate how power and autonomy directly influenced motivation to engage in weight‐related discussions, shaping whether conversations were perceived as supportive or coercive.

### 4.3 The Need for Personalisation and Collaboration

Both groups identified a lack of personalisation as a barrier to meaningful discussions about weight. Standardised policies were perceived as imposed rather than negotiated, limiting opportunities for meaningful dialogue. For example, uniform restrictions on sugar intake were perceived as dismissive of personal differences:

Some people are bigger, some people are smaller, why should…everybody be put under the same rule…deal with the person more individually (SU2)

From the staff perspective, rigid, one‐size‐fits‐all guidelines were also described as constraining conversations. Nurses reported that such policies limited their ability to engage in nuanced discussions about weight, particularly where individual medical needs varied. In contrast, personalised care planning was seen as providing a legitimate and clinically defensible framework for initiating and sustaining dialogue:

a care plan would be good to be personalised to their own needs, and nurses can support them (NS10)

Collaborative goal‐setting further facilitated sustained engagement in weight‐related discussions. Service users described how jointly agreed goals created continuity in conversations, enabling progress to be revisited without judgement and fostering a sense of shared ownership:

if you haven't achieved that goal, you still know that it's there, [it keeps] me motivated …[you see] how far you have come (SU15)

These findings show that personalised, collaborative approaches were central to sustaining engagement in weight‐related discussions over time.

Overall, the results demonstrate that weight‐related discussions in secure mental health inpatient settings were shaped by the interaction of staff capability, organisational opportunity and relationally mediated motivation. When discussions were early, collaborative and embedded within relational safety, engagement was facilitated. Where communication was delayed, authoritarian or constrained by organisational pressures, discussions were avoided or curtailed.

## 5 Discussion

This study illuminates the complex interplay of capability, opportunity and motivational factors that shape the initiation of and engagement in weight‐related discussions between nursing staff and service users within one UK low‐ and medium‐secure mental health service. Consistent with previous work highlighting the elevated risk of cardiometabolic illness in forensic and secure populations (Johnson et al. 2018; NHS England 2024), both groups recognised the importance of addressing physical health. However, within this service, engagement was strongly influenced by how weight‐related conversations were initiated and how power, safety and readiness were negotiated within the therapeutic relationship, highlighting that the conduct of these discussions may be as important as their content.

The findings suggest that barriers and enablers extended beyond individual attitudes, reflecting relational and organisational features of this secure care context. For nursing staff, capability‐related factors were central: confidence and skill in behaviour‐change communication acted as enablers, but these were frequently undermined by limited training in motivational strategies and uncertainty about navigating sensitive topics without triggering distress or conflict. This gap aligns with prior research identifying persistent workforce development limitations in physical health promotion and behavioural change competencies among mental health nurses (Attala et al. 2022; Davies et al. 2023; Mateo‐Urdiales et al. 2020). Environmental pressures, including high acuity care demands, staffing shortages, monotonous environments and incident‐driven ward routines, further reduced opportunity, shifting practice from proactive health promotion to a reactive, risk‐driven model, as previously observed in forensic inpatient services (McCullough 2023).

From the service users' perspective, capability‐related barriers were pronounced. Cognitive difficulties, limited nutritional knowledge and illness‐ or medication‐related challenges constrained participants' ability to engage with information and apply advice consistently. This mirrored findings in existing literature (Moss et al. 2025). Service users emphasised the need for scaffolded, accessible education and ongoing support rather than on–off guidance, echoing prior evidence on the variability of physical health engagement at admission (Huthwaite et al. 2017).

Across both groups, relational dynamics emerged as a prominent influence on whether weight‐related discussions were possible, welcomed or avoided. Staff and service users described weight as a high‐risk topic, emotionally, clinically and professionally. Power imbalances shaped perception: staff feared complaints or allegations (Attala et al. 2022), while service users described concerns about judgement or infantilisation (Holley et al. 2020). Within this setting, these mutual anxieties created a delicate relational environment in which the tone, timing and structure of conversations were critical. Staff role modelling was an additional enabler; visible engagement in healthy behaviours was perceived to enhance credibility, whereas inconsistent or contradictory practices undermined trust and motivation. This is consistent with existing literature suggesting that positive staff role modelling may support health‐promoting behaviours (Kelly et al. 2017).

In keeping with the Theoretical Domains Framework (TDF) (Atkins et al. 2017), these findings illustrate how capability, opportunity and motivation interact to shape behaviour (Cane et al. 2012). Within this service, staff capability influenced whether discussions were initiated; organisational and environmental opportunity determined whether these conversations could be sustained; and motivational factors, including service users' intrinsic enjoyment of health behaviours and extrinsic goals such as weight or health benefits, affected engagement and adherence (Albarracín et al. 2024). Importantly, personalised, collaborative goal‐setting and autonomy‐supportive communication emerged as critical for sustaining dialogue. In secure settings, where autonomy is inherently constrained (Pedersen et al. 2025), these strategies preserve agency and foster therapeutic alliances, consistent with recovery‐oriented practice and self‐determination theory.

Within this service, weight management conversations are not purely informational or procedural. Rather, they are complex, relationally embedded behaviours that require alignment between staff skills, ward culture and service user readiness. These findings suggest that approaches addressing all three domains, strengthening staff capability, maximising opportunities through supportive, engaging weight‐related discussions and fostering motivation through personalised, collaborative approaches, are likely to improve the initiation, sustainability and effectiveness of weight‐related discussions.

Taken together, the findings support consideration of co‐designed, recovery‐focused approaches that embed weight management within routine nursing care. Prioritising relational safety, personalised goal‐setting and staff capability may provide a theoretically grounded pathway to support engagement and meaningful physical health promotion within this and potentially comparable secure mental health settings (Markham 2021). Further research across different secure services is needed to examine the transferability of these findings and how contextual factors influence weight‐related discussions.

### 5.1 Strengths and Limitations

A key strength of this study is the inclusion of both nursing staff and service users, allowing for triangulation of perspectives and a relational analysis of how weight‐related discussions are initiated and experienced in secure mental health settings. Use of the COM‐B framework enhanced analytic rigour and supported theoretically informed interpretation of factors shaping engagement.

The study was conducted in a single NHS low and medium‐secure service in the UK, which may limit direct transferability to international settings where legal frameworks, level of security and models of care differ. However, the focus on communication, relational safety and autonomy‐supportive practice offers conceptually transferable insights relevant to other secure and forensic contexts. High secure services were not included due to their distinct security arrangements and service users' profiles, which may influence weight‐related discussions differently and warrant separate investigation.

## 6 Conclusion

This study illustrates how weight‐related conversations within one UK low‐ and medium‐secure mental health service were shaped by interacting capability, opportunity and relational dynamics. Staff capability influenced whether conversations were initiated, with limited confidence and training in behaviour‐change communication contributing to avoidance or risk‐managed, task‐focused discussions. Opportunities to engage were shaped by environmental pressures, including staffing levels and ward acuity, which constrained continuity and depth of dialogue. Critically, relational dynamics, particularly perceptions of power, autonomy and emotional safety, also appeared to shape whether weight‐related discussions were experienced as collaborative and supportive or as intrusive and coercive.

Within this setting, meaningful engagement in weight‐related discussions appeared to depend on two interconnected conditions: staff feeling skilled and supported to initiate sensitive conversations, and service users experiencing these conversations as personalised, autonomy‐supportive and embedded within trusting therapeutic relationships. Where these conditions were absent, discussions were fragmented, reactive and often limited to policy enforcement rather than shared understanding. In contrast, early and routine conversations, framed collaboratively and linked to individual goals, appeared to create opportunities for ongoing dialogue and sustained engagement. Further research across different secure mental health services is needed to examine the transferability of these findings and how contextual factors influence practice.

The findings suggest that, within this service, weight management may not be adequately supported through information‐giving or standardised policies alone. A co‐designed, recovery‐oriented approach that prioritises personalised and collaborative conversations may better support engagement while respecting dignity and autonomy. Future research across diverse secure mental health settings could examine how staff communication capability, organisational opportunity and relational safety interact to support engagement in weight‐related discussions and weight management.

## 7 Relevance to Practice

Although this study was conducted in one UK secure mental health service, this study suggests that weight management within this setting is a relational practice shaped by trust, autonomy and ward culture (Bennett and Hanna 2021; Moss et al. 2022). These findings are consistent with international literature, suggesting that early, routine and collaborative discussions embedded within care planning may normalise engagement and reduce perceived risk (McCullough 2023). Strengthening staff communication skills may support confidence in initiating sensitive conversations (Happell et al. 2023; Hurley et al. 2022). Personalised, co‐designed approaches may further support motivation, engagement and autonomy in weight management (Shewen and Adams 2024; Stewart 2024).

## Author Contributions

All authors have made substantial contributions to the conception and design of the study, data collection, analysis and interpretation, and the drafting and revision of the manuscript. All authors have read and approved the final version of the manuscript and agree to its submission for publication.

## Funding

The authors have nothing to report.

## Conflicts of Interest

The authors declare no conflicts of interest.

## Data Availability

The data that support the findings of this study are available from the corresponding author upon reasonable request.
